# A New Recursive Trigonometric Technique for FPGA-Design Implementation

**DOI:** 10.3390/s23073683

**Published:** 2023-04-02

**Authors:** Xing Xing, Wilson Wang

**Affiliations:** 1 Department of Electrical and Computer Engineering, Lakehead University, Thunder Bay, ON P7B5E1, Canada; xxing2@lakeheadu.ca; 2 Department of Mechanical Engineering, Lakehead University, Thunder Bay, ON P7B5E1, Canada

**Keywords:** FPGA, trigonometric functions, CORDIC, LUT, digital signal processing

## Abstract

This paper presents a new recursive trigonometric (RT) technique for Field-Programmable Gate Array (FPGA) design implementation. The traditional implementation of trigonometric functions on FPGAs requires a significant amount of data storage space to store numerous reference values in the lookup tables. Although the coordinate rotation digital computer (CORDIC) can reduce the required FPGA storage space, their implementation process can be very complex and time-consuming. The proposed RT technique aims to provide a new approach for generating trigonometric functions to improve communication accuracy and reduce response time in the FPGA. This new RT technique is based on the trigonometric transformation; the output is calculated directly from the input values, so its accuracy depends only on the accuracy of the inputs. The RT technique can prevent complex iterative calculations and reduce the computational errors caused by the scale factor *K* in the CORDIC. Its effectiveness in generating highly accurate cosine waveform is verified by simulation tests undertaken on an FPGA.

## 1. Introduction

In modern electronic systems, trigonometric functions are commonly used in satellite communication, 5G mobile communication, system control, digital signal processing, etc. [1]. Several embedded platforms, such as the Field-Programmable Gate Array (FPGA) and application-specified integrated circuitry, can help implement trigonometric functions in electronic systems [2]. The lookup table (LUT) [3], polynomial approximation [4], and coordinate rotation digital computer (CORDIC) [5,6,7,8,9] are the main algorithms for implementing trigonometric functions in these embedded platforms. Among these algorithms, although the LUT has small latency, it requires a large storage capacity of the ROM [10]. The polynomial approximation has higher accuracy, but it requires more complex multiplications and square operations [11]. In addition, although CORDIC is a commonly used algorithm to implement trigonometric functions in embedded systems, it still has drawbacks in the actual design [11,12,13,14,15,16,17,18,19,20]. For example, even though a high-radix CORDIC such as 4-radix, 16-radix, or hybrid CORDIC can reduce the iteration and simplify the process procedure, its computation of the variable scale factor is very time-consuming [17]. Although the scaling-free CORDIC can avoid complex calculations, its convergence and accuracy are limited [1].

On the other hand, the scale-free CORDIC designed in [1,6] can enhance convergence and reduce power consumption and latency through booth recoding; however, its inherent CORDIC iteration remains unchanged, which will increase the complexity of the CORDIC algorithm. Hence, a novel algorithm is essentially needed to implement trigonometric functions so as to offer a streamlined approach and improve the computation accuracy in contemporary electronic systems.

To tackle the aforementioned problems, a new recursive trigonometric (RT) technique will be proposed in this work to provide a simpler but more accurate analytical model forcalculating trigonometric function values. The RT technique is inspired by recursive methods in computer programming, which can be used to divide a problem into several sub-problems, solve each of those sub-problems, and then synthesize the results of these sub-problems. The recursion method breaks the factorial into the product of the base input and the recursive calculation. Once the input value is defined, the recursive chain keeps running until the program ends [21]. The proposed RT technique is new in the following aspects: (1) It employs trigonometric identities such as Angle Addition and Subtraction to elucidate the relationships between the sine and cosine functions. Different from the CORDIC algorithm, the RT technique employs a streamlined computational model to improve the accuracy of trigonometric function generation. (2) The new RT technique will calculate the values of trigonometric functions directly so as to prevent delays associated with multiple iterations and reduce execution time on FPGAs. The efficacy of the RT technique will be assessed through simulation tests.

## 2. Recursive Trigonometric Technique

In this Section, the RT technique and its implementation strategy on FPGA are discussed.

### 2.1. Principle of the RT Technique

The proposed RT technique is based on the trigonometric identities to conduct trigonometric function calculation. It is motivated by the fact that the cosine functions can be calculated easily using other trigonometric functions (e.g., sine, tangent, and cotangent) by the trigonometric identities. Equations (1) and (2) are the basic cosine and sine function expansions:

(1) cos((n+1)θ)=cos(θ)cos(nθ)−sin(nθ)sin(θ)

(2) $$\sin(n\theta )\sin(\theta )=\frac{1}{2}\left[\cos(n\theta -\theta )-\cos(n\theta +\theta )\right]$$

Based on Equations (1) and (2), the following representation can be obtained:

(3) $$\cos\left((n+1)\theta \right)=\cos(\theta )\cos(n\theta )-\frac{\cos(n\theta -\theta )-\cos(n\theta +\theta )}{2}$$

Equation (3) can be simplified as

(4) cos((n+1)θ)=2cos(θ)cos(nθ)−cos((n−1)θ)

The RT technique will be derived from the trigonometric identities transform in Equation (4), specifically:

If *n* = 1,

(5) cos(2θ)=2cos(θ)cos(θ)−cos(0)

If *n* = 2,

(6) cos(3θ)=2cos(θ)cos(2θ)−cos(θ)

…

If *n* = *m*,

(7) cos(mθ)=2cos(θ)cos((m−1)θ)−cos((m−2)θ)

From Equations (5) to (7), it can be seen that given an initial angle *θ*, we can calculate the values of cos(*θ*), cos(2*θ*), …, cos(*mθ*), recursively, where *m* is an integer. Therefore, once the initial angle is selected, all cosine values of cos(*mθ*) can be computed, mθ∈[0, 2π]. To generate an entire periodic cosine signal, the RT technique is essentially a pipelined computational process. Once the initial value has been entered, the calculation of the cosine value can be executed. The computation result will be utilized directly in the subsequent cosine calculation, and so forth. The accuracy of the RT technique is also different from the traditional CORDIC because computation is affected by the accuracy of the input cosine values only, but not by the iterations and rotation coefficients.

### 2.2. FPGA Architectures of the RT Technique

In the RT technique, the trigonometric calculation will cover each cosine angle over [0, 2π]. The initial angle cos(0) and the iteration step angle cos(*θ*) should be selected properly based on applications. For example, the iteration step size of the angle can be selected so that the following input will be the cosine value of the selected step size angle without a non-integer number of iterations. The iteration step can be an integer angle in degrees, a fractional angle, or an angle in radians, but the angle value should be an integer multiple of 2π rad or 360 degrees.

**Figure 1:**
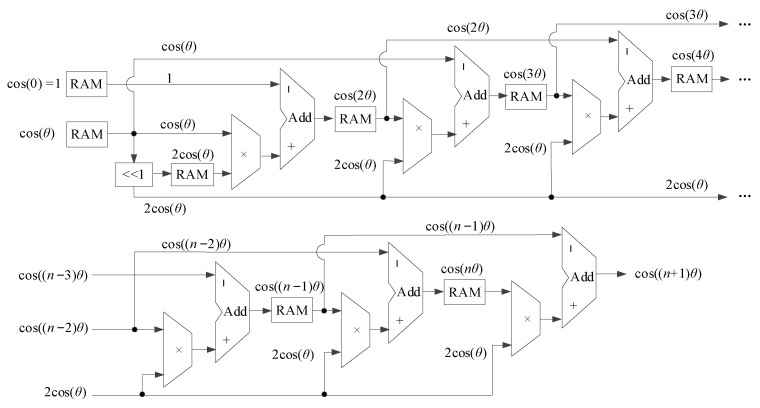
The architecture of the RT algorithm.

Figure 1 shows the digital architecture to implement the RT technique to calculate the cosine values. Firstly, store the initial angle and iteration step angle in the RAM. The shifter will shift the step size angle to the left or multiply by 2, resulting in 2cos(*θ*); it will then be multiplied by cos(*nθ*) according to Equation (5). Next, 2cos(*θ*)cos(*nθ*) will be subtracted in the accumulator, which generates 2cos(*θ*) and is stored in the RAM for the following calculations. The output accuracy depends on the precision of the input angle only, whereas the input angle precision relies on the bit resolution of the device or system in the application.

## 3. Simulation Test and Analysis

Some simulations will be undertaken in this section to use the RT technique to generate the cosine waveforms. The RT technique will be implemented in MATLAB and ModelSim. The tests will be undertaken on an FPGA platform. The RT’s effectiveness will be examined by comparing its performance with the related methods under the same testing conditions.

### 3.1. MATLAB Simulation and Analysis

**Figure 2:**
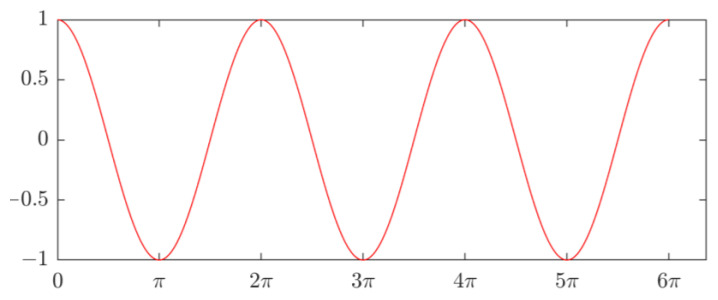
The RT cosine waveform simulation by MATLAB.

Figure 2 shows simulated cosine waveforms in MATLAB using the RT technique. The step angle is 0.006 rad used for the RT, radix-2 CORDIC, and radix-4 CORDIC, respectively. The scale factor *K* = 0.607 is used for radix-2 CORDIC with 16 iterations [13]. The scale factor *K* is a variable for radix-4 CORDIC, with eightiterations [13]. For the RT technique, theinitial value is the cosine value of the step angle: cos(0.006). The cosine value of the step angle and cos(0)are used to compute the following cosine values recursively using Equation (5). The result precision will keep in a 16 bits binary format for the computation of each algorithm.

**Figure 3:**
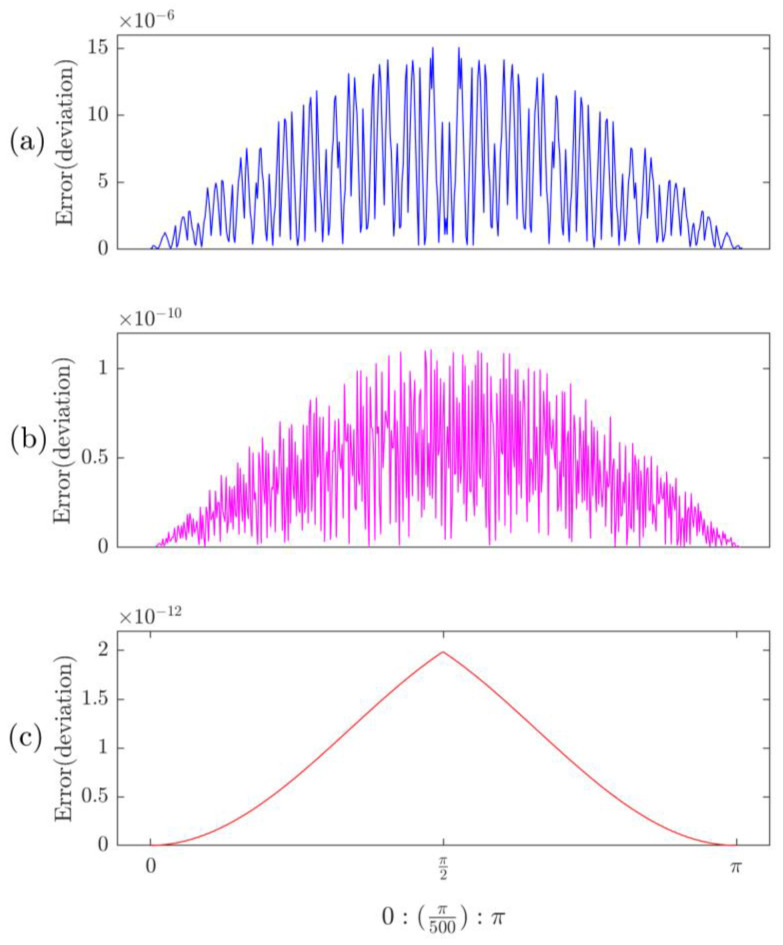
16-bit accuracy comparison among different methods: (**a**) Radix-2, (**b**) Radix-4, (**c**) RT.

Figure 3 shows the 16-bit comparison among these algorithms. It can be seen that the maximum CORDIC difference error occurs at π/2 for the radix-2 (Figure 3a) and radix-4 (Figure 3b), respectively. Because the CORDIC has angles only over (−1.74, +1.74) rad or (−99.99, +99.99) degrees, based on tan(*θ*), it can calculate two quadrants’ angles only. The angle out of this interval can be converted into (−1.74, +1.74) rad or (−99.99, +99.99) degrees. As a result, calculation errors will increase as more iterations are undertaken.

The scale factor *K* will affect the accuracy of CORDIC. The radix-2 CORDIC is an approximate calculation algorithm; its final cosine value is multiplied by a scale factor of approximately 0.607 as the number of iterations reaches infinity. For the radix-4 CORDIC, the scale factor *K* is not a constant [16] but can be calculated by

(8) $$K=\prod_{i}{\left(1+\sigma_{i}^{2}\times 4^{-2i}\right)}^{1/2}$$

where $\sigma_{i}$ belongs to the digit set {−a,…,0,…,+a}, a∈[2, 3]; *i* is the number of iterations; when *i* achieves *n* bits, the result precision is *n*/2.

$\sigma_{i}$ can be determined by angle intervals, as discussed in [16]. Different angles in eachiteration will result in a different $\sigma_{i}$ value. Although radix-4 CORDIC can decrease the iteration time, its scale factor calculation is more complex compared with radix-2 using Equation (8).

**Figure 4:**
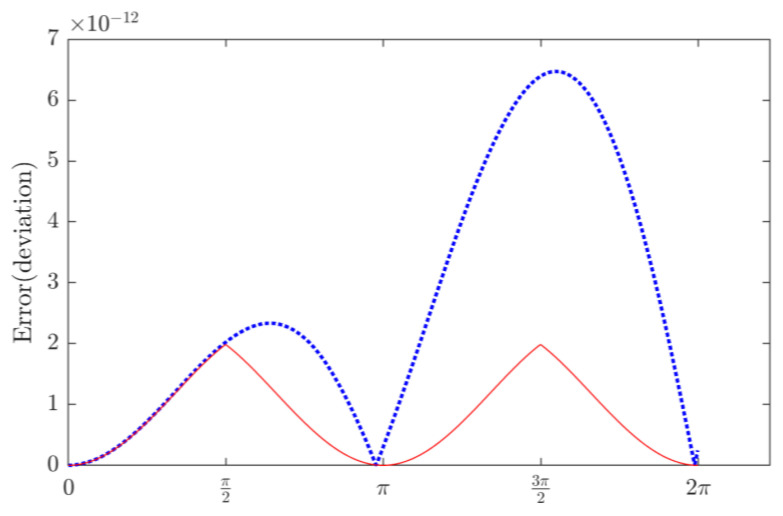
Accuracy comparison of the RT algorithms using and without using the quadrant transformation, represented by a solid line and a dashed line, respectively.

As illustrated in Figure 3c, the proposed RT technique generates the maximum error of 1.98 × 10^−12^, which is much lower than the radix-2 (1.50 × 10^−5^) and radix-4 (1.10 × 10^−10^) algorithms, as shown in Figure 3a and Figure 3b, respectively. For the RT technique, the processing errors mainly come from two sources: (1) the pre-define cosine value; (2) the accumulated truncation errors in the recursive calculation. The former error can be reduced by using more accurate input cosine values such as 24 bits or 32 bits in binary format. The latter error can be reduced by using quadrant transformation. The applied angle domain is (0, π/2), and the angles beyond that range can be transformed to (0, π/2). For example, the value of cos(3π/2) can be transformed to cos(π/2). To illustrate the improvement in the accuracy of the RT algorithm by applying quadrant transformation, Figure 4 offers a comparison using the RT algorithms with and without using the quadrant transformation.

As shown in Figure 4, the RT algorithm without using quadrant transformation generates the maximum error of 6.47 × 10^−12^ around 6.46 rad and the second maximum error peak of 2.34 × 10^−12^ around 1.98 rad. Since the truncation error cannot be eliminated over (0, π), the error is continuously accumulated over (π, 2π).In contrast, the RT algorithm using the quadrant transformation computes cosine values only over the (0, π/2) domain, which can prevent the error accumulation in computing cosine values beyond π/2. Its maximum error is 1.98 × 10^−12^ at 1.56 rad, which is much lower than 6.47 × 10^−12^ generated by the RT algorithm without applying the quadrant transformation. Or the quadrant transformation can avoid the accumulation of truncation errors so as to improve the overall accuracy of the RT technique.

To further examine the effectiveness of the proposed RT technique in calculation accuracy, some comparison tests are undertaken using MATLAB in terms of the root mean square error (RMSE). Table 1 summarizes respective errors with 16-bit and 32-bit precision of RT and other related methods such as CORDIC II [11] and Hybrid CORDIC [13].

It is seen from Figure 3 and Table 1 that the proposed RT technique outperforms other related algorithms in precision due to its quadrant transformation. Each cosine value is directly calculated based on the recursion in Equation (5). On the other hand, the CORDIC and its related improved methods, such as Hybrid CORDIC and CORDIC II, are approximation algorithms; the calculation accuracy of their cosine values depends on not only the iterations but also the selection of the scale factor *K*.

### 3.2. ModelSim Simulation and Analysis

The effectiveness of the proposed RT technique will be further examined in accuracy and flexibility by some simulation tests on the ModelSim environment.

In initialization, the step angle of 0.088 rad is selected, and the bandwidths of the cosine results are 16 bits and 32 bits. The CORDIC will use 16 and 32 iterations with a scale factor of *K* = 0.6072. The RT technique will use the same step angle and also take 16 bits and 32 bits to make the test conditions compatible with those used in CORDIC and LUT. The cosine value of 0.542 rad is used as a reference for comparison. Table 2 summarizes the resulting cosine values using these three methods.

As observed from Table 2, both the RT and LUT algorithms outperform the CORDIC algorithm in terms of accuracy at 16 bits. This is because the CORDIC relies on the scale factor *K* and the number of iterations, which will degrade its cosine calculation accuracy. Utilizing restricted 16 data bits, the RT technique can produce the highest precision approximate cosine value, which is equivalent to the accuracy offered by the LUT. In 32-bit analysis, although the LUT generates the highest accuracy (2.04 × 10^−10^), it requires a significant volume of data that must be pre-stored in memory (90 cosine values in this case). On the other hand, the accuracy of the RT technique is 10 times higher than the CORDIC in the 32 bits (1.47 × 10^−9^ vs. 1.43 × 10^−8^). Therefore, the RT technique provides the best comprehensive performance in comparison with the LUT and CORDIC algorithms. Table 3 summarizes the comparison with different bandwidths of the same angle cosine value by using the RT technique.

The RT is a recursive algorithm by which the cos((*n* + 1)*θ*) is computed based on cos(*θ*) and cos((*n* − 1)*θ*) values. Therefore, more accurate cos(*θ*) values can generate more accurate computations of the following trigonometric functions. As illustrated in Table 3, if the bandwidth increases from 8 bits to 32 bits, the RT can compute a 0.524 rad cosine value with much higher accuracy (7.68 × 10^−2^ vs. 1.47 × 10^−9^), or the accuracy increases exponentially.

It is seen from Equation (5) that the accuracy of the RT mainly depends on two factors: the step angle cosine value and the truncation error arising from the use of multiplication in the calculation process, as discussed in Section 2.2. The step angle cosine value is expressed in a constant 32-bit binary format. In the course of the processing stage, ensuring a consistent computational bandwidth requires the execution of multiplication through a rightward data shift. This operation subsequently contributes to the emergence of truncation errors. Consider an example in Table 4, if the sampling step angle is 0.088 rad, the computation of the 1.484 rad cosine value, cos(1.484), will take 17 RT recursions in comparison with 6 RT calculations for 0.542 rad cosine value, cos(0.542), in Table 3. Therefore, the cos(1.484) value has a lower accuracy than the cos(0.542) value because of the accumulated truncation errors. In addition, it is seen from Table 4 that the accuracy of the RT technique depends on the bandwidth of the application. A wider bandwidth will provide a higher accuracy in RT processing.

On the other hand, it is seen from Table 3 and Table 4 that the RT generates different cosine values in different bits device platforms. Unlike the LUT algorithm, the RT technique does not need to pre-store each of the required angle values in the ROM, while the value of cos(*θ*) is the only stored quantityfor the following waveform generation and processing. The RT algorithm can use any initial angle to calculate the waveforms, which can facilitate software programming and hardware implementation.

Table 5 summarizes the RT technique for latency comparison. The test environment is to generate a full cosine function of 16-bit width at a 50 MHz system clock. The CORDIC and its improved methods use seveniterations or more for shifting, whereas the RT uses only one iteration to generate the required cosine values, which can significantly reduce the calculation and processing time. It is seen from Table 5 that the RT technique takes only 80 ns to complete the calculation of cosine functions, which is much faster than other related techniques.

### 3.3. FPGA Implementation of Cosine Functions

**Figure 5:**
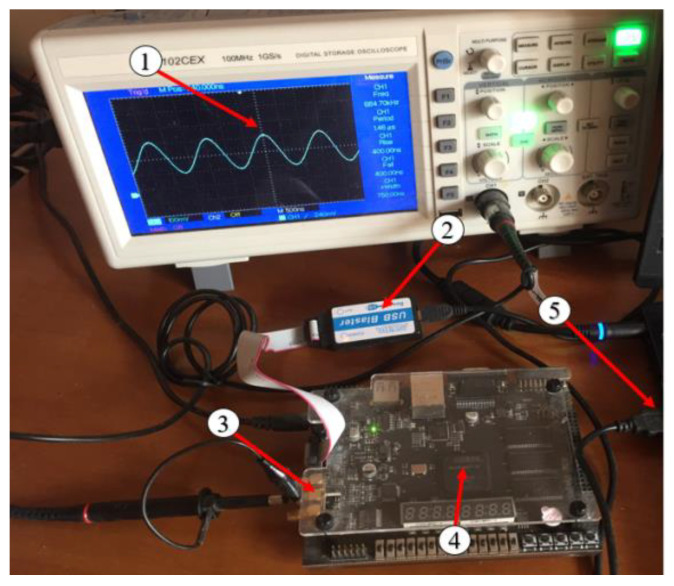
Experiment setup of FPGA implementation of the cosine waveform: (1) Oscilloscope; (2) FPGA USB blaster; (3) DAC output; (4) Cyclone IV E FPGA; (5) Connection to a PC.

**Figure 6:**
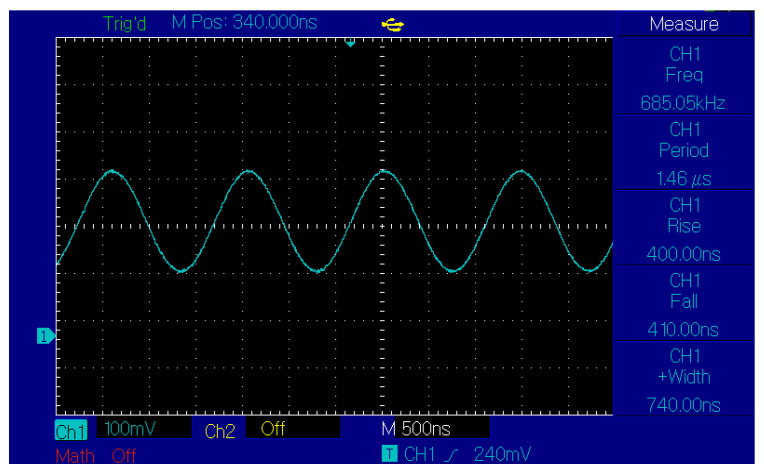
A cosine waveform generated by Cyclone IV FPGA using the RT technique.

The RT technique is coded in Verilog, synthesized using Quartus software, and implemented on the Cyclone IV E FPGA (EP4CE15F23C8 from Intel, San Jose, CA, USA). The digital output is converted to an analog signal using a DAC (AD9707 from Analog Device Inc, Wilmington, NC, USA). The bandwidth of DAC input is 12 bits. The outputs from the DAC are scaled so that the first and second bits are used for the plus/minus sign bit and decimal point bit, respectively. Figure 5 shows the experiment setting, and Figure 6 demonstrates the generated cosine waveforms using the RT technique.

The initial angle of 0.086 rad is selected such that a complete cosine cycle requires generating 73 cosine values. The input clock of the FPGA I/O pins is 50 MHz, or the period is 20 ns. Then the cycle frequency of the final DAC output will be

(9) $$f_{OUT}=\frac{1}{73\times 20\times 10^{-9}}=684,931$$

This can be recognized in Figure 6. The experimental results are fully consistent with the theoretical results, which can verify the feasibility of using the RT technique on the FPGA. The test results using the RT technique and other related algorithms in terms of resource and power consumption are summarized in Table 6 and Table 7, respectively.

It is seen from Table 6 that the RT technique is more resource-intensive than CORDIC due to its use of multipliers that consume a large number of logic units. However, this issue could be mitigated by designing and using a special hardware multiplication unit. In addition, like CORDIC, the RT technique does not require a large number of memory cells to store data as LUT-based approaches. Furthermore, as demonstrated in Table 7, the proposed RT technique uses the lowest power consumption among the related algorithms.

## 4. Conclusions

A recursive trigonometric, RT, technique has been proposed in this work to provide a new approach for FPGA implementation of trigonometric functions. The RT technique leverages the inherent properties of trigonometric functions to compute the target cosine value using the input cosine value directly. Consequently, the precision of the processing result is commensurate with the accuracy of the input value; it can circumvent the need for complicated calculation procedures and avoid the errors that may arise from the rotation factor. Its effectiveness has been examined by simulation tests. Test results have shown that the proposed RT technique can provide high accuracy in computation, a simple structure in implantation, and high efficiency in processing. It has the potential forwide applications such as digital synchronizers, waveform generators, and communication systems. Specifically, from ModelSim simulation, the RT technique outperforms other related algorithms (with 1 × 10^1^ orders higher in precision) at 16-bit and 32-bit bandwidths, as well as better performance in latency due to its straightforward computation approach. From simulation tests on the Cyclone IV E FPGA device, the RT technique has demonstrated its better performance in resource and power consumption. It has the potential forreal-world applications such as digital synchronizers, waveform generators, and communication systems. On the other hand, the RT technique has the following possible limitations: (1) the processing accuracy could vary with input value accuracy; (2) it could still have accumulated truncation errors in calculation; and (3) it has resource usage due to multipliers. Advanced research is undertaken to enhance the hardware by incorporating FPGAs with dedicated hardware multipliers, improve its processing speed and accuracy, as well as verify its efficiency in the actual physical platforms.

## Figures and Tables

**Table 1:** Comparison of different algorithms to generate cosine waveform.

Algorithm	16 Bits RMSE	32 Bits RMSE
Radix-2 [22]	1.39 × 10^−4^	1.69 × 10^−6^
Radix-4 [16]	6.85 × 10^−5^	1.07 × 10^−6^
CORDIC II [11]	8.70 × 10^−3^	N/A
Hybrid [13]	1.70 × 10^−5^	N/A
RT	2.82 × 10^−9^	1.02 × 10^−12^

**Table 2:** Accuracy comparison of different algorithms to generate cosine waveform.

Algorithm	16 Bits	Deviation	32 Bits	Deviation
CORDIC	56,769	2.32 × 10^−4^	929,887,710	1.43 × 10^−8^
LUT	56,755	1.48 × 10^−5^	93,719,550,786	2.04 × 10^−10^
RT	56,755	1.48 × 10^−5^	5,929,887,683	1.47 × 10^−9^

**Table 3:** Comparison of different bandwidth 0.524 rad cosine value generated by the RT technique.

Bandwidth	Result	Deviation
8 bits	220	7.68 × 10^−2^
16 bits	56,755	1.48 × 10^−5^
32 bits	929,887,683	1.47 × 10^−9^

**Table 4:** Comparison of different bandwidth 1.484 rad cosine value generated by the RT technique.

Bandwidth	Result	Deviation
8 bits	−18	1.81
16 bits	5537	2.70 × 10^−2^
32 bits	93,582,683	8.87 × 10^−7^

**Table 5:** Comparison of latency using the related algorithms.

Algorithm	Iterations	Time (ns)
Radix-2 CORDIC [22]	16	360
Radix-4 CORDIC [16]	11	320
Hybrid [13]	7	160
CORDIC II [11]	7	140
RT	1	80

**Table 6:** Comparison of the resource consumption using the related algorithms.

Algorithm	Logic Elements	ROM (Bits)
CORDIC	2363/15,408 (12%)	0
LUT	24/15,408 (<1%)	4096
RT	2677/15,408 (17%)	0

**Table 7:** Comparison of the power consumption using the related algorithms.

Algorithm	Dynamic (mW)	Static (mW)	Total (mW)
CORDIC	14.13	49.63	90.93
LUT	0	49.29	88.39
RT	10.52	49.47	87.58

## Data Availability

Data unavailable.
